# The safety and quality of pork and poultry meat imports for the common European market received at border inspection post Hamburg Harbour between 2014 and 2015

**DOI:** 10.1371/journal.pone.0192550

**Published:** 2018-02-09

**Authors:** Wiebke Jansen, Svenja Woudstra, Anja Müller, Nils Grabowski, Gundela Schoo, Bettina Gerulat, Günter Klein, Corinna Kehrenberg

**Affiliations:** 1 Institute for Food Quality and Food Safety and Research Center for Emerging Infections and Zoonoses (RIZ), University of Veterinary Medicine Hannover, Foundation, Hannover, Germany; 2 Border Inspection Post Hamburg Harbour, Civil Authority for Health and Consumer Protection, Hamburg, Germany; University of Campinas, BRAZIL

## Abstract

Though imports of products of animal origin into the European Union (EU) have to comply with legal requirements and quality standards of the community, food consignment rejections at external EU borders have been increasing in recent years. This study explored microbiological metrics according to national target and critical values valid for samples at consumer level of 498 fresh poultry meat and 136 fresh pork filets from consignments subjected to physical checks during clearing at the border inspection post Hamburg harbour between January 2014 and December 2015 with ISO standard methods. Quantitative results indicated that critical thresholds for aerobic counts, *Enterobacteriaceae*, and *E*. *coli* were never surpassed. Merely for staphylococci, one poultry sample (0.2%) and 10 pork samples (9.3%) exceeded the critical limit (3.7 log cfu/g). However, qualitative analyses revealed that, *Staphylococcus aureus* was present in 16% and 10% of all poultry and pork samples, respectively, though no methicillin-resistant *Staphylococcus aureus* could be confirmed. Moreover, *E*. *coli* was present in 50% and 67% of all pork and poultry samples, respectively, and thereof 33 isolates were confirmed as extended-spectrum β-lactamase-producing *E*. *coli*. Only 1.2% of the poultry samples were unacceptable due to the presence of *Salmonella* spp., whereas they were not detected in any pork sample. *Campylobacter* spp. were not detected in any sample. Though imported pork and poultry meat complies mostly with national market requirements, it might pose a potential risk to public health, especially for a direct or indirect foodborne transmission of imported, uncommon strains of zoonotic bacteria.

## Introduction

The European Union (EU) is the second biggest importer of food worldwide and the EU seeks to guarantee that all imports fulfil the same high standards as products from EU member states (MS) with respect to food hygiene but also regarding the animal health status [1]. Consequently, import of products of animal origin (POAO) into the EU MS from non-EU countries underlies specific conditions and rules. Regarding trading partners abroad, non-EU countries as well as the single food business operator within the respective country must be authorised for the introduction of a specific category of POAO into the EU, fulfilling all community requirements. Approved non-EU countries authorised for the introduction of meat are listed officially in accordance with all legal requirements [2]. During clearance of consignments at EU entry points, official veterinarians at designated EU border inspection posts (BIP) therefore check all documentation and the identity of every single consignment arriving for legal compliance before these enter the EU territory. The frequency of physical checks depends on the risk profile of the product and also on the results of previous checks. In particular, physical checks are mandatory for at least 20% of consignments containing fresh meat and meat products (including offal of bovine, ovine, caprine, porcine and equine species) and 50% of poultry meat and poultry meat products [3]. Consignments found not to be compliant with Community legislation shall be rejected at all EU BIPs.

In recent years, notifications on these border rejections have been increasing exponentially, particularly due to high detection rates of *Salmonella* spp. in consignments of poultry meat and products thereof, whereas red meat consignments such as pork and beef are most often rejected due to findings of shiga-toxin-producing *Escherichia* (*E*.) *coli* (STEC) [4]. However, those microbiological analyses are not performed systematically. The strict preconditions for legal imports specified by the supranational authorities are rather performed risk-based, either on suspicious consignments or under the umbrella of the Multi Annual National Control Plan (MANCP). They cover feed and food, animal health and animal welfare controls in each MS [5]. Therefore, studies on the microbial quality and safety of legally imported pork and poultry meat into the EU are exceptional and most often focused on the detection of antimicrobial-resistant bacteria. Zogg et al. [6], reported that 100% and 66.7% of imported poultry meat from Argentina (n = 2) and Brazil (n = 3) at retail in Switzerland harboured extended β-lactamase (ESBL)-producing *Enterobacteriaceae*, but methicillin-resistant *Staphylococcus* (*S*.) *aureus* (MRSA) were only detected in EU-produced meat. Egervärn et al. [7] reported ESBL or transferable AmpC beta-lactamase (pAmpC)-producing *E*. *coli* at a high prevalence (95%) in poultry meat imported predominantly from Brazil (n = 40), but also from Argentina and Chile (n = 3) to Sweden.

Despite the lack of scientific publications, those baseline data are essential parts of risk analyses and crucial for food safety authorities to reassure and verify veterinary standards on food quality and safety. Import risk analysis has been implemented mandatorily by the World Trade Organisation (WTO) by means of its sanitary and phytosanitary measures agreement (SPS Agreement). To avoid unjustified barriers to trade, imported products have to be treated non-discriminatorily and not less favourably than domestically-produced goods. The SPS Agreement dictates that all measures must be scientifically based and not unnecessarily restrictive. This requires improved surveillance and monitoring systems, risk analysis capabilities and quality assurance, and lastly adequate laboratory investigations of imported products [8].

To the best of our knowledge, the present paper is the first attempt to describe baseline microbial metrics of a large, representative sample size of legally imported fresh pork and poultry meat and salted meat preparations. We explored the prevalence of major food safety metrics such as *Salmonella*, *Campylobacter* and *Yersinia* but as well quality criteria such as the total aerobic mesophilic bacteria count, *E*. *coli* and *Enterobacteriaceae*, and staphylococci. In addition, the prevalence of ESBL-producing *E*. *coli* and MRSA were screened.

## Material and methods

### Sampling

In 2014 and 2015, the BIP Hamburg harbour received a total of 260 and 310 consignments of fresh poultry meat and 1,629 and 1,518 poultry meat preparations, respectively. In contrast, fresh pork consignments amounted for only 85 consignments in 2014 and 96 in 2015. All consignments were, however, cleared and subjected to the obligatory physical control check [3]. During these physical checks in the BIP laboratory, a minimum of 250g per consignment was taken aseptically and stored at -18°C in labelled and sealed plastic bags to ensure traceability. The frozen samples were transferred quarterly to the Institute of Food Quality and Food Safety, Hannover (Germany) for further analyses. All samples were kept frozen at -18°C during storage and transport. In total, n = 718 poultry and n = 20 pork samples were collected by the competent authorities at the BIP Hamburg harbour in the years 2014 and 2015.

Only fresh meat and salted meat preparations (poultry breast and leg meat without any other seasoning, additives or ingredients) were considered suitable for microbiological analyses. Fresh meat was defined as meat that has not undergone any preserving process other than chilling, freezing or quick-freezing, including meat that was vacuum-wrapped or wrapped under a controlled atmosphere. Meat preparations were defined as uncooked fresh meat, including meat that has been reduced to fragments, which has had foodstuffs, seasonings, or additives added to it or which has undergone processes insufficient to modify internal muscle fibre structure of the meat and therefore eliminate the characteristics of fresh meat [9]. In order to increase the pork sample size, we included n = 119 commercially available Chilean frozen, boneless pork filets from a major German cash-and-carry wholesaler located in Hannover (Germany). The pork filets had been cleared via the BIP Hamburg Harbour but were not subjected to physical control.

### Microbiological analysis

A subset of 516 poultry meat and 136 pork samples were subjected to microbiological analyses. Preparation of the frozen samples was performed in accordance with ISO 6887–2:2003 [10], followed by performing ISO standard methods for microbiology in the food chain. Due to technical issues of the sampling process at the BIP Hamburg harbour, 205 samples were excluded from further analyses. Criteria were: (i) less than 250g in total, (ii) slices with less than 2cm thickness, (iii) more than 4 single pieces per individual sample. After analyses, all samples were properly disposed of in accordance with legal regulations [11].

In total, four criteria were evaluated quantitatively: (i) total aerobic colony counts (ACC) were assessed in accordance with DIN EN ISO 4833–2:2013 [12] and that of (ii) *Enterobacteriaceae* were investigated in accordance with DIN EN ISO 21528–2:2004 [13]. The enumeration of (iii) *E*. *coli* was performed in accordance with DIN EN ISO 16649–2:2001 [14]. Presumptive *E*. *coli* were streaked on ESBL Brilliance Agar (Oxoid, Wesel, Germany) to screen for the presence of ESBL/AmpC-producing *E*. *coli*. Presumptive ESBL/AmpC *E*. *coli* were confirmed and characterized by antimicrobial susceptibility testing, multilocus sequence typing (MLST), macrorestriction analysis, microarray analysis and additional PCR assays according to Müller et al., 2017 [15]. The enumeration of (iv) *Staphylococcus* spp. was performed in accordance with DIN EN ISO 6888–1:1999 [16]. Subsequent to the identification of *S*. *aureus*, presumptive positive colonies were streaked on ChromID MRSA Agar (Biomerieux, Marcy-l’Etoile, France) to screen for methicillin/oxacillin resistant isolates. Six presumptive methicillin-resistant *S*. *aureus* were confirmed and characterized by antimicrobial susceptibility testing, *spa* typing, MLST, macrorestriction analysis, microarray analysis, and *dru* typing according to Müller et al., 2016 [17]. However, quantitative microbiological analyses have detection limits, and to reduce the risk of classifying samples as false-negative, additional qualitative analyses were performed for *Enterobacteriaceae*, *E*. *coli* and staphylococci. After enrichment in NaCl-Peptone water (Oxoid, Wesel, Germany) for 24 ± 2h in 37 ± 0.5°C, samples were streaked fractionated on selective agar and incubated in accordance with each specific ISO standard method. Results were interpreted considering the corresponding ISO standards.

In addition, three criteria were evaluated only by qualitative analysis. The detection of (i) *Salmonella* was performed in accordance with DIN EN ISO 6579:2002 with an initial enrichment of a 10g sample [18]. The detection of (ii) thermophilic *Campylobacter* spp. with an initial enrichment of a 10g sample was performed exclusively for poultry meat in accordance with DIN EN ISO 10272–1:2006 [19], whereas the detection of (iii) *Yersinia enterocolitica* presumed to be pathogenic to humans was performed exclusively for pork in accordance with ISO 10273:2003 with an initial enrichment of a 10g sample [20].

### Evaluation criteria

Microbiological criteria on community level are laid down merely for fresh poultry meat with a zero tolerance in 25g regarding *Salmonella* serovar Typhimurium and *Salmonella* serovar Enteritidis for products placed on the market during shelf life [21]. However, the German Society of Hygiene and Microbiology (DGHM), Hannover (Germany) published further recommendations on target and critical values of microbiological criteria valid at consumer level. Samples were evaluated against these given limits of the DGHM for samples at retail level. Criteria are considered as (i) satisfactory if the log_10_ of colony forming units (cfu) per gram was below the target value (TV); (ii) acceptable if the log_10_ cfu was between the TV and the critical value (CV) and (iii) unsatisfactory if the log_10_ exceeded the CV. A zero tolerance was applied for *Salmonella* spp. (Table 1).

**Table 1:** DGHM microbial quality and safety criteria for fresh pork and poultry meat.

	Poultry meat (fresh)		Pork (fresh)	
	Target value	Critical value	Target value	Critical value
Aerobic colony count (ACC)	6.7 log_10_ cfu/g	NA	6.7 log_10_ cfu/g	NA
Enterobacteriaceae	4 log_10_ cfu/g	5 log_10_ cfu/g	4 log_10_ cfu/g	5 log_10_ cfu/g
*E*. *coli*	2.7 log_10_ cfu/g	3.7 log_10_ cfu/g	2 log_10_ cfu/g	3 log_10_ cfu/g
*Staphylococcus* spp.	2.7 log_10_ cfu/g	3.7 log_10_ cfu/g	2.7 log_10_ cfu/g	3.7 log_10_ cfu/g
*Salmonella*	Absence in 25g		Absence in 25g	

NA = not applicable

Microbiological data were compiled and entered into a spreadsheet. Data were analysed and processed in Microsoft ^®^ Excel 2011 and RStudio. Data were visualised in Excel and Adobe Illustrator®.

## Results

### Pork filet

A total of n = 136 fresh Chilean boneless pork filets were evaluated that have been cleared between 01.01.2014 and 31.12.2015.

#### Quantitative analyses

**Fig 1:**
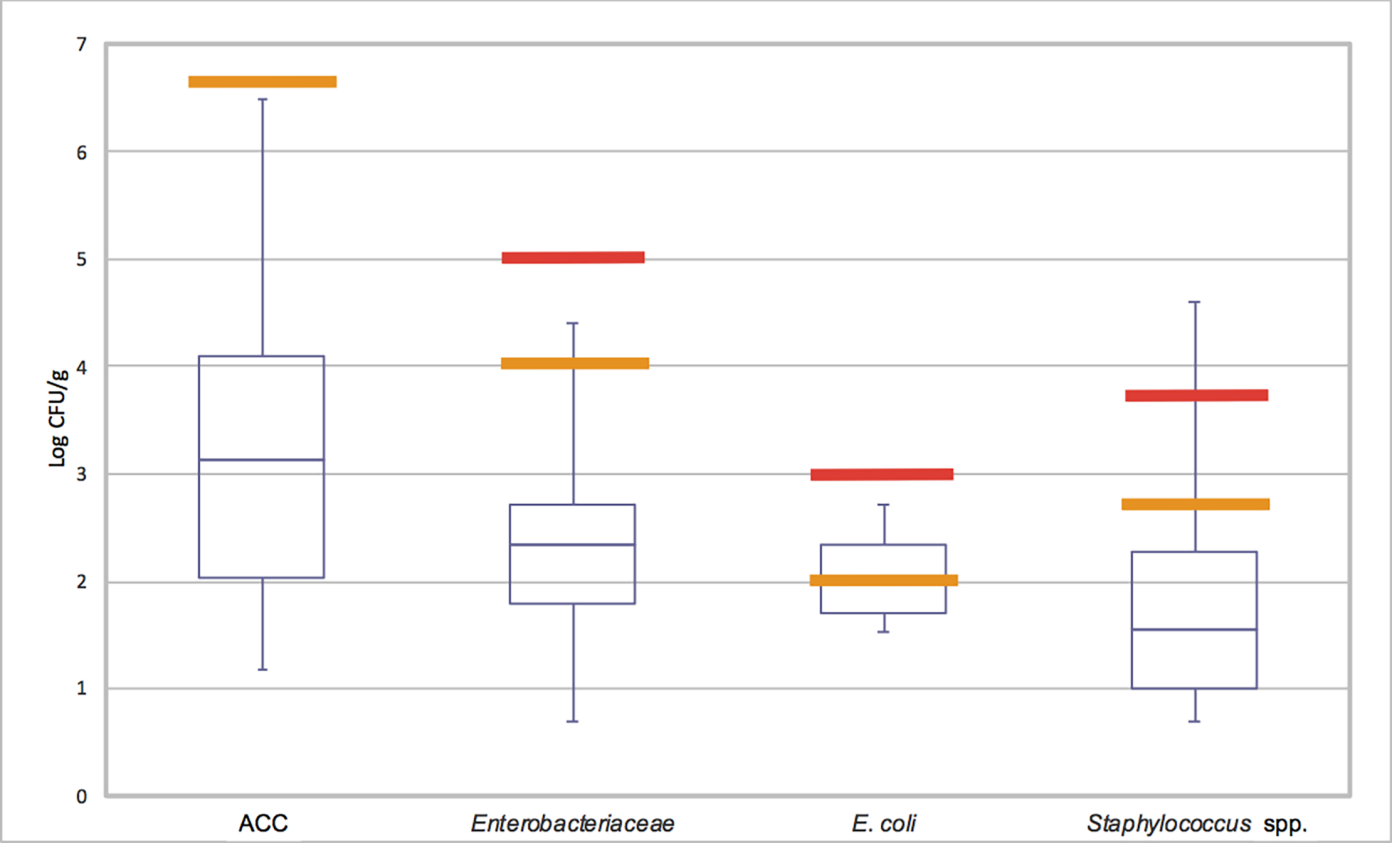
Boxplot of the mean concentration of four microbiological criteria (Aerobic colony count (ACC), *Enterobacteriaceae*, *E*. *coli* and staphylococci) assessed for 136 Chilean pork filets cleared by the BIP Hamburg harbour in the years 2014 and 2015. Yellow bold bars indicate the target value, red bold bars indicate the critical value to classify quality categories.

The mean concentration in all samples of the total ACC amounted to 3.2 log_10_ cfu/g, of *Enterobacteriaceae* to 2.45 log_10_ cfu/g, and of staphylococci to 1.78 log_10_ cfu/g, thus, being far below the legal thresholds. In contrast, the mean counts of *E*. *coli* were 2.04 log_10_ cfu/g, exceeding the target value as recommended by the DGHM. However, the range of the fourth quartile was wide for the ACC, *Enterobacteriaceae* and staphylococci, being lesser in the case of *E*. *coli*. The boxplots shows, that the determination of each respective first quartile was self-limiting due to the detection limit of the first dilution in the preparation of the samples [10]. The majority of individual pork filet samples were classified as satisfactory as the log_10_ of colony-forming units (cfu) per gram was below the target value (TV). A minority was classified as acceptable for *Enterobacteriaceae* (n = 13, 9.6%), *E*. *coli* (n = 10, 7.4%) and staphylococci (n = 8, 8,6%) as the log_10_ cfu ranged between the TV and the critical value (CV). Ten samples (7.4%) were unsatisfactory for staphylococci, surpassing the CV of 3.7 log_10_ cfu/g (Fig 1).

#### Qualitative analyses

No samples were classified as unacceptable due to the presence of *Salmonella*, neither was *Yersinia enterocolitica* detected in any sample.

The qualitative analysis after enrichment indicated *Enterobacteriaceae* in 96% (n = 121) of the samples and *E*. *coli* in 50% (n = 63) of the samples, thereof one confirmed ESBL-producing *E*. *coli* [15]. Staphylococci were detected in 99% of all samples; thereof 10.3% (n = 13) were confirmed as *Staphylococcus aureus*. According to Müller et al. [17], no MRSA was confirmed in further analyses

### Poultry meat

A total of 498 fresh poultry meat samples were evaluated between 01.01.2014 and 31.12.2015. A number of 18 samples were excluded from evaluation due to technical concerns during the analyses. The majority of samples (n = 426 [86%]) were chicken meat, the remaining samples were turkey meat (n = 72 [14%]), however both originated primarily from Brazil (n = 300 chicken meat [71%], n = 61 turkey samples [85%]). The remaining turkey meat originated from Chile (n = 10 [14%]) and one single turkey meat sample was received from Israel. Chicken meat samples originated second most frequently from Thailand (n = 86 chicken meat [20%]), followed by Chile (n = 39 [9%]) and one single chicken meat sample was received from Argentina.

#### Quantitative analyses

**Fig 2:**
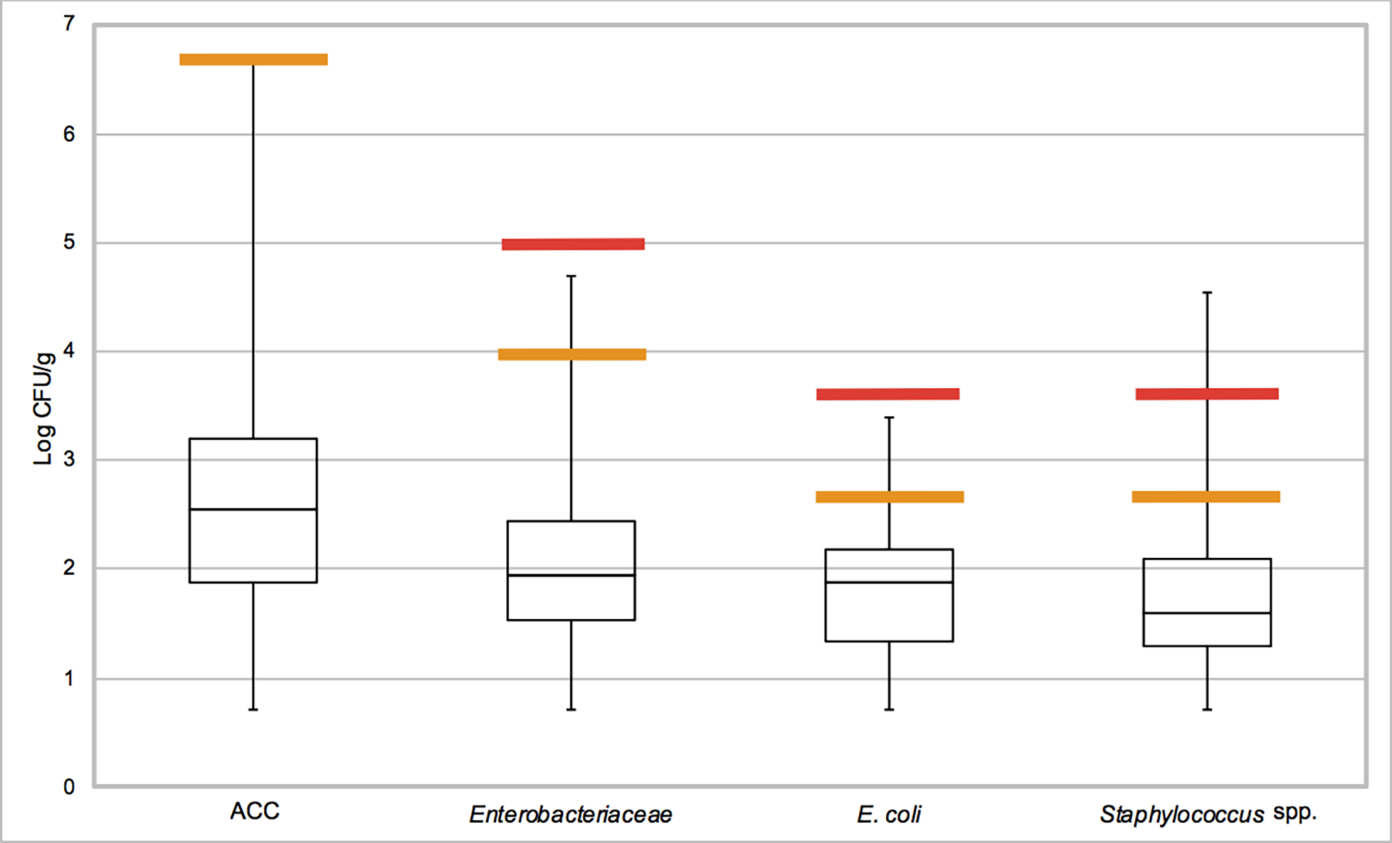
Boxplot of the mean concentration of four microbiological criteria (Aerobic colony count (ACC), *Enterobacteriaceae*, *E*. *coli*, and staphylococci) in 498 poultry meat consignments cleared by the BIP Hamburg harbour in the years 2014 and 2015. Yellow bold bars indicate the target value, red bold bars indicate the critical value to classify the quality categories.

In the poultry samples, the mean ACC were 2.58 log_10_ cfu/g, 2.08 log_10_ cfu/g of *Enterobacteriaceae*, 1.82 log_10_ cfu/g of *E*. *coli* and 1.7 log_10_ cfu/g of staphylococci. However, the analysis of the boxplot shows that the range of each respective fourth quartile was wide, whereas the respective first quartile was limited. This indicates a considerable variation, though the detection limit owing to the first dilution in the preparation of the samples is self-limiting [10]. The mean counts were still far below the legal thresholds. Regarding all poultry samples, the vast majority of individual samples was classified as satisfactory as the log_10_ of colony forming units (cfu) per gram was below the TV. Less than 1% was classified as acceptable for *Enterobacteriaceae*, *E*. *coli* and staphylococci, as the log_10_ cfu was between the TV and the CV. One single sample was unsatisfactory for staphylococci, surpassing the CV of 3.7 log_10_ cfu/g. Fig 2 shows the resulting microbiological evaluation.

**Fig 3:**
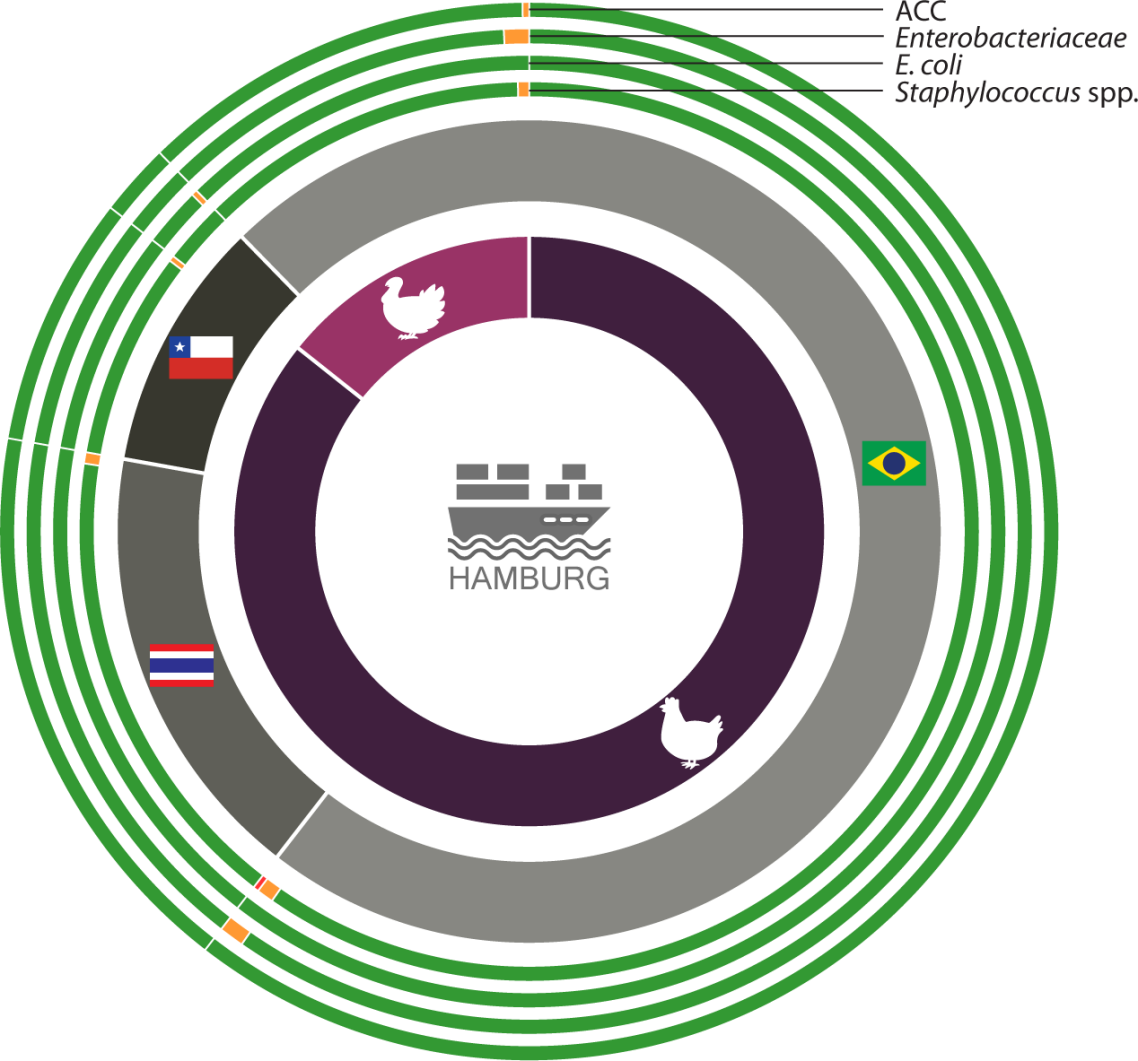
Classification of fresh poultry meat according to the species (inner circle: chicken and turkey) and country of origin (central circle: Brazil, Thailand and Chile) in three quality categories in accordance with microbiological criteria of the DGHM in per cent [%] (outer circle): (1) satisfactory (green); (2) acceptable (yellow), and (3) unsatisfactory (red).

In particular, three and four chicken meat samples from Brazil surpassed the TV for *Enterobacteriaceae* and *Staphylococcus* spp., respectively. Moreover, one chicken sample from Brazil surpassed even the CV for staphylococci. The vast majority of samples from Thailand and Chile were satisfactory in terms of legal thresholds, only one and two samples, respectively, surpassed the CV for *Staphylococcus* spp. The single chicken meat sample from Argentina was satisfactory in terms of legal thresholds but qualitative analyses revealed that it contained *S*. *aureus*. The turkey meat originating from Chile complied well with legal thresholds; only one sample was classified as unacceptable for *E*. *coli*. (14% of n = 72). In contrast, all turkey meat samples from Brazil complied with legal *E*. *coli* thresholds, but surpassed the CV for ACC in n = 1, *Enterobacteriaceae* in n = 4, and for staphylococci in n = 2 samples. The single turkey meat sample received from Israel complied with all legal thresholds (Fig 3).

#### Qualitative analyses

A percentage of 1.2% (n = 6) samples was classified as unsatisfactory due to the presence of *Salmonella* spp. *Campylobacter* spp. were not detected in any sample.

In good accordance to the quantitative analyses, the qualitative analysis after enrichment showed a prevalence of *Enterobacteriaceae* in 99% (n = 497) of the poultry samples, and of *E*. *coli* in 67% (n = 339) of the samples. Of these, 32 ESBL-producing *E*. *coli* were identified. Staphylococci were detected in 95% (n = 475) of the samples; thereof 16% (n = 78) were confirmed as *S*. *aureus*. In particular, the single chicken meat sample from Argentina contained *S*. *aureus*, even though it satisfactorily complied with legal thresholds. Furthermore, *S*. *aureu*s was detected disproportionally often in samples from Chile (n = 10, 26% detection rate) and Thailand (n = 20, 23% detection rate) compared to samples from Brazil (n = 45, 15% detection rate). Moreover, one turkey meat sample each from Chile (10% detection rate) and Brazil (1.6% detection rate) contained *S*. *aureus*. However, and despite the differences in percentages, no significant differences of the independent variables (origin and type of sample) were indicated by a logistic regression on the detection rates of *S*. *aureus* for chicken nor for turkey meat between the different countries (confidence interval of 95%). None of the *S*. *aureus* was confirmed to be MRSA [17].

## Discussion

EU citizens have high expectations regarding the safety and quality of food, regardless the origin of the product. The present paper reports the microbial quality and safety status of legally imported meat from third countries into the EU, including the presence of ESBL-producing *E*. *coli* and MRSA in.

### Quality of imported pork and poultry meat

The vast majority (>99%) of poultry samples complied with the ACC market requirements. The ACC is a general microbiological indicator for food quality, particularly for the maintenance of the cold chain as the ACC represents the total amount (cfu) of mesophilic microorganisms. We received only frozen samples, and due to the satisfactory results, we can conclude that the storage during transport was in accordance with the legal regulations. Only smaller percentages, 9.6%, 7.4% and 13.4% of all pork filets surpassed the satisfactory target value for *Enterobacteriaceae*, *E*. *coli* and staphylococci, respectively. *Enterobacteriaceae* and *E*. *coli* are indicators of water or food quality and their presence may be an indication of unhygienic processing condition. In our study, *E*. *coli* was the most frequent microbial contamination detected on poultry in 67% and on pork in 50% of all samples. Pathogenic *E*. *coli* usually lead to gastro-intestinal symptoms including fever, diarrhoea and abdominal cramps. In particular, shiga-toxin-producing entero-pathogenic *E*. *coli (*STEC) harbour the potential to cause serious harm to consumers as an infection can lead to the haemolytic uremic syndrome (HUS) in humans, kidney failure and may be fatal. EFSA reported, in 2015, a total of 15 positive samples from 296 single samples and 12 batches (4.9%). In addition, a total of 84 samples from turkey meat and 609 from broiler meat were tested with only five STEC O157-positive samples (0.8%) in broilers [22]. Even though we did not test for the major STEC virulence genes *stx* and *eae*, the high proportion of contaminating *E*. *coli* in our samples indicates a poor hygiene status and a possible infection source. Whilst microbiological contamination and other hazards are omnipresent in meat, culinary tradition in Europe includes the consumption of undercooked meat such as pork filet and raw meat products, e.g. short ripened pork sausages, posing a considerable public health risk. Contaminated carcasses may play an important role in the direct and indirect transmission of zoonotic *E*. *coli*. Moreover, ESBL-producing *E*. *coli* constitute an additional concern since the initial treatment of invasive infections with these isolates is often inappropriate, resulting in an increased mortality rate in affected patients [23]. It was shown that EU meat imports might harbour various *E*. *coli* strains different from those commonly known in Europe. Particularly broiler meat from South America with a prevalence of 95% (38/40 samples) ESBL/AmpC-producing *E*. *coli* was considered as a potential source to human exposure [7]. With 32 ESBL/AmpC-producing *E*. *coli* from 498 South American poultry samples (6.4%), we detected much less than previously reported by screening the very large sample size [15].

The critical value of staphylococci was rarely surpassed, with only 0.2% of poultry, but 7.5% of pork samples. *S*. *aureus* on meat may indicate contamination at pre-harvest or the presence of cross-contamination with human body discharges. High contamination of food with *S*. *aureus* is generally due to improper personal hygiene during handling and processing. A further analysis on staphylococcal toxins of all encountered strains would have been necessary to assess the food safety risk of these samples, but laid beyond the designated framework of this study. Bacterial toxins, including those from *Staphylococcus* spp., were the third most frequent cause for foodborne outbreaks in the EU in 2013 [24]. Moreover, the contamination with *S*. *aureus* poses an additional risk for consumers, being present in 16% of poultry and 10% of all pork samples of our study. Molecular analyses of this subset in the course of a subsequent study revealed the presence of genes encoding important virulence factors, such as the toxic shock syndrome toxin-1 and different enterotoxins [17]. A recent study supports our findings on the prevalence of *S*. *aureus* in Chilean retail pork, albeit found at a higher percentage (51.8%) whilst detecting various antibiotic resistance profiles, including multi-drug resistance [25]. However, none of our samples contained molecularly confirmed MRSA [17].

### Safety of imported pork and poultry meat

In 2015, *Salmonella* spp. continued to be the second most commonly reported gastrointestinal bacterial pathogens in humans in the EU and the pathogen is most frequently detected in poultry meat [22]. In accordance with the DGHM criteria, *Salmonella* spp. must be absent in a 25g sample of the product when placed on the market and also during shelf life. In our study, neither pork nor turkey meat, but 1.2% (n = 6) of individual chicken meat samples were classified as unacceptable due to the presence of *Salmonella* spp. This is much less than in domestic products. EFSA reported non-compliance in domestic broiler meat due to *Salmonella* spp. in 6.5% of the 16,981 units tested in 2015. In particular, *Salmonella* spp. were found in 5.3% of single samples (rising up from 2.2% in 2014) and in 5.7% of batches (lowering from 9.5% in 2014) [22]. The overall proportion of *Salmonella*-positive broiler meat samples at retail was 7.4%, which was higher than at the slaughterhouse (6.3%) and at the processing plant (6.7%) levels. Moreover, EU MS tested a total of 47,038 units of fresh pig meat in 2015, of which 1.7% were *Salmonella*-positive, mainly at slaughterhouse level. In comparison, in 2014, a total of 68,134 units of pig meat were examined, and 0.5% were *Salmonella*-positive [24]. In our study, four *Salmonella*-positive samples originated from Brazil and two from Thailand. A study from Brazil, the principal exporter of broiler meat to the EU, reported a mean prevalence of *Salmonella* spp. of 2.7% (range 0.0%-8.9%), primarily found in the State of São Paulo (50.6% of positive samples). The data originated from the period from September 2004 to July 2006, when 2,679 frozen chicken carcasses at retail from 15 Brazilian cities were examined [26]. However, it should be noted that the EU domestic poultry market offers basically chilled products only [27], whereas freezing and the long storage time below -18°C induces in *Salmonella* a viable but not-culturable (VNBC) state, in which they remain or regain their virulence but are not detectable with classic cultural methods [28]. This may contribute to our low detection rates.

Furthermore, we did not detect any *Campylobacter* spp., even though broiler meat is considered as the most important single source of human campylobacteriosis. In 2015, a high (46.7% of 6,707 tested units) prevalence of *Campylobacter* spp. in EU domestic fresh broiler meat was reported by 14 MS in samples at slaughter, processing and retail, including single and batch samples from all sampling stages [22]. It was confirmed recently that *Campylobacter* spp. are essentially temperature-sensitive and feature a VBNC state under unfortunate environmental conditions [28]. As all samples were received frozen, this may account for the absence of cultural detection. Though *Campylobacter* spp. detection in food is notifiable in 12 EU MS, including Germany, at present there is no harmonized surveillance, sampling strategies, nor detection methods for *Campylobacter* spp. in the EU. Some MS collect more samples during the high-prevalence summer period and thus do not report an overall annual prevalence. Therefore, monitoring results are not comparable between reporting countries and years [22].

Though five EU MS reported 5.4% *Yersinia*-positive findings in domestic pig meat and products thereof from retail [24] (mainly *Y*. *enterocolitica*), none was detected in our study. It is known that pork and particularly pork filet is generally less contaminated due to its protected position within the carcass compared to the tonsils which is the main site of detection [29].

### The EU market of imported poultry and pork meat

Food safety and quality is nowadays of global priority and it is imperative for every country to ensure compliance and appropriate testing of their products regarding their trade status. Only few countries comply with EU regulations, but accordingly hold specific agreements on veterinary sanitary measures regarding trade with the EU. For meat and meat products from all species, countries of origin must be on the positive list of eligible countries for the product [2]. Despite its self-sufficient production of pork (111%) and poultry meat (103%), the EU imported, in 2014, 0.8 Mio t high-value poultry products, such as poultry breasts and cooked preparations etc., mainly from Brazil (60% of total EU poultry meat imports) and from Thailand (30%) [30]. Currently, several countries, such as Argentina, Brazil, Canada, Chile, Israel, the Russian Federation, Thailand, Ukraine and the United States of America may export fresh poultry meat and meat preparations to the EU. Poultry meat imports amounted to more than 870 thousand tonnes in 2013. Imports are expected to grow gradually from 2013 to 2014, approaching 1 million t by 2025, as a consequence of increased production in two of the EU’s main supplier countries, Thailand and Brazil [27]. Undoubtedly, trade flows depend strongly on long-established trade relations, most favourable shipping routes, and waterways. For example, poultry imports to Germany mainly originate from Brazil, while Chile is the major trading partner of the United Kingdom [31].

In contrast, the EU import quantities of pork accounted for 17 to 20 thousand tonnes in the past decade and are expected to remain within the range of 20 thousand t in the coming years [32]. The EU pork trade is restricted to only four countries, namely Australia, Chile, Canada and the United States of America. All pork filets in our study were of Chilean origin. This mirrors recent import activities; only 3,200 t pork were imported to Germany in 2014, mainly from Chile. In particular, Chilean pork imports even dropped to 2,000 t (-27%) compared to 2013. Moreover, pork import quantities remain negligible as the European pork market is currently oversaturated with a self-sufficiency of 111% of the inner-EU production [33]. Therefore, these changes in EU pork consumption patterns may limit domestic demand and in particular the concerns of import from non-European countries may aggravate the trade situation. This situation was also reflected in difficulties in obtaining a sufficient sample size in the course of the present study.

## Conclusions

International trade improves the global availability of products of animal origin but enables foodborne zoonotic and multi-resistant bacteria to spread worldwide. Trade agreements and legal regulations on the import of products of animal origin are well implemented and provide the highest level of food safety for the EU consumers. Therefore, all imports of fresh meat and meat products into the European Union are subject to veterinary certification, but microbial analyses thereof are rare. We confirm that only minor quantities of poultry meat are unacceptable due to microbiological contamination, whereas pork does surpass legal thresholds to a considerable amount. Even though the vast majority of imported consignments comply with market requirements, imported poultry and pork might pose a potential risk to public health for a direct or indirect foodborne transmission of zoonotic bacteria, and in particular those resistant to antimicrobials, due to the consumption of and cross-contamination with raw or undercooked meat.
